# Bifrost: highly parallel construction and indexing of colored and compacted de Bruijn graphs

**DOI:** 10.1186/s13059-020-02135-8

**Published:** 2020-09-17

**Authors:** Guillaume Holley, Páll Melsted

**Affiliations:** Faculty of Industrial Engineering, Mechanical Engineering and Computer Science, University of Iceland, Reykjavík, Iceland

## Abstract

Memory consumption of de Bruijn graphs is often prohibitive. Most de Bruijn graph-based assemblers reduce the complexity by compacting paths into single vertices, but this is challenging as it requires the uncompacted de Bruijn graph to be available in memory. We present a parallel and memory-efficient algorithm enabling the direct construction of the compacted de Bruijn graph without producing the intermediate uncompacted graph. Bifrost features a broad range of functions, such as indexing, editing, and querying the graph, and includes a graph coloring method that maps each *k*-mer of the graph to the genomes it occurs in.

**Availability**

https://github.com/pmelsted/bifrost

## Introduction

The de Bruijn graph is an abstract data structure with a rich history in computational biology as a tool for genome assembly [1, 2]. With the advent of high throughput sequencing, the Overlap Layout Consensus (OLC) framework frequently used to assemble Sanger sequencing data [3] was progressively replaced in favor of de Bruijn graph-based methods. Since 2008, a wide range of genome assemblers based on the de Bruijn graph have been released [4–10]. Although single molecule sequencing technologies [11, 12] have re-introduced the OLC framework as the method of choice to assemble long and erroneous reads [13–16], de Bruijn graph-based methods are nonetheless used to assemble and correct long reads [17, 18]. Overall, de Bruijn graphs have found widespread use for a variety of problems such as de novo transcriptome assembly [19], variant calling [20], short read compression [21], short read correction [22], long read correction [17], and short read mapping [23] to name a few. The colored de Bruijn graph is a variant of the de Bruijn graph which keeps track of the source of each vertex in the graph [24]. The initial application was for assembly and genotyping, but it has also found use in pan-genomics [25], variant calling [26], and transcript quantification methods [27].

Despite serving as a building block for many methods in computational biology, the de Bruijn graph adoption is hindered by two factors. First, the memory usage and computational requirements for building de Bruijn graphs from raw sequencing reads are considerable compared to alignment to a reference genome, while only a handful of tools have focused on de Bruijn graph compaction [28–33]. Second, de Bruijn graph construction usually requires tight integration with the code. In the best case, software libraries for building and manipulating de Bruijn graphs are used [34, 35], but in most cases, data structures to index the de Bruijn graph are re-implemented. Those downsides are intensified in the colored de Bruijn graph for which the memory consumption of colors rapidly overtakes the vertices and edges memory usage [36]. For this reason, a lot of attention has been given to succinct data structures for building the colored de Bruijn graph [30, 31, 36–41] and data structures for multi-set *k*-mer indexing [42–47]. In the following, we focus on tools for constructing compacted de Bruijn graphs (cdBGs) with or without colors. We refer the reader to the survey of [48] for more details about *k*-mer-based data structures as well as the reviews of [25] and [49] for data structures to index collections of *k*-mer sets.

TwoPaCo [28] is a highly parallel construction tool for the cdBG. It builds progressively the cdBG from assembled genomes by identifying *junctionk*-mers which are either branching or located at the extremities of unitigs. A Bloom filter is first used to approximate the graph and a hash table is subsequently employed to remove false positives. The approach taken by BCALM2 [29] is orthogonal to the one of TwoPaCo: rather than identifying junction *k*-mers, BCALM2 incrementally assembles *k*-mers into unitigs until junction *k*-mers are reached. *K*-mers are partitioned according to their minimizers, and partitions are compacted independently in parallel. A final step glues the compaction of different partitions together. Note that BCALM2 can process assembled genomes as well as short read data. deGSM [50] performs an external sorting of the *k*-mers from the input sequences and then constructs a Burrows-Wheeler transform (BWT) [51] of the unitigs from which the final graph is extracted. SplitMEM [30] uses the suffix tree [52] to construct a ccdBG. Unitigs of the graph are derived from the set of Maximum Exact Matches in the input genomes, while colors are implicitly encoded in the suffix tree. SplitMEM is not adapted to short read data input and splits the unitigs to ensure all *k*-mers of each unitig share the same set of colors. Baier et al. [31] provided two algorithms improving SplitMEM with a lower time complexity using a Compressed Suffix Tree and the BWT. PanTools [33] creates first an uncompacted *k*-mer index from which are derived unitigs. By iterating over the input assembled genomes, *k*-mers that have not been visited yet are extended to form unitigs, possibly leading to the merging and splitting of previously created unitigs. The graph index is maintained in a database providing edit operations such as updating the graph with additional data. PanTools was specifically designed for pan-genomic applications with assembled genomes in input and allows gene annotations in the graph.

In this paper, we present Bifrost, a software for efficiently constructing, indexing, and querying the colored and compacted de Bruijn graph (ccdBGs), both in terms of runtime and memory usage. The data structures and algorithms implemented in Bifrost are specifically tailored for fast and lightweight construction, querying, and dynamic manipulation of compacted de Bruijn graphs, both regular and colored. The software is designed to take advantage of multiple cores and modern processors instruction sets (SIMD operations). Bifrost is also available as a C++11 software library with minimal external dependencies and allows developers to build on top of an efficient de Bruijn graph engine by using the Bifrost API. Bifrost has been successfully employed for alignment- and reference-free phylogenomics [53] as well as bacterial genomes querying of genes linked to pathogenicity islands and *fluoroquinolone* resistance [54].

## Results

We benchmarked Bifrost against state-of-the-art software on publicly available dataset. We focus on three representative use cases: cdBG construction, cdBG querying, and cdBG coloring. All experiments were run of a server with an 16-core Intel Xeon E5-2650 processor and 256G of RAM. Running time was measured as wall clock time using the time command, and peak memory was measured by ps.

### cdBG construction

We constructed the cdBG of the NA12878 human genome short read dataset from the Genome In A Bottle consortium [55]. The dataset is downsampled from 300-fold to 30-fold coverage to reflect normal sequencing depth, resulting in about 696 million 150-bp paired-end sequences.

We compared Bifrost to BCALM2 because of its low computational requirements and versatility as it can build a cdBG from short read data or assembled genomes. BCALM2 can be configured for different memory usage where a lower memory usage results in a longer running time. In our experiments, it was configured with the maximum memory usage of Bifrost for each *k*-mer size tested. Additionally, BCALM2 uses by default up to 5 GB of disk space while Bifrost does not use any disk except for the final output. Results are shown in Table 1, and summaries of the unitig N50, *k*-mer cardinality, and unitig cardinality in each graph built are reported in Table 2.

**Table 1:** Time and memory comparison of Bifrost and BCALM2 for different *k*-mer sizes and number of threads during graph construction

	Tool	*k*-mer size	Number of threads			
			1	4	8	16
Time (h)	Bifrost	31	**20.81**	**8.53**	**6.10**	**5.55**
		63	**14.38**	**4.20**	**2.40**	**2.00**
		95	**12.51**	**3.88**	**2.25**	**1.58**
		127	**9.56**	**2.96**	**1.81**	**1.41**
	BCALM2	31	44.25	14.11	8.48	6.33
		63	N/A	25.6	13.96	8.71
		95	N/A	39.91	21.45	12.56
		127	N/A	N/A	27.73	16.15
Memory (GB)	Bifrost	31	39.59	39.58	39.59	39.60
		63	37.77	37.77	37.77	37.78
		95	44.33	44.30	44.30	44.32
		127	55.88	55.86	55.86	55.86
	BCALM2	31	**36.00**	**35.66**	**35.61**	**35.58**
		63	N/A	**29.83**	**29.73**	**29.64**
		95	N/A	**33.47**	**33.51**	**33.66**
		127	N/A	N/A	**43.42**	**53.77**

Best results are highlighted. N/A indicates the result is unavailable because the computation took more than 48 h

**Table 2:** Unitig N50, *k*-mer, and unitig cardinalities in cdBGs built from NA12878 for different *k*-mer sizes

*k*-mer size	*k*-mer cardinality	Unitig cardinality	Unitig N50
31	2,675,559,250	80,478,269	421
63	2,991,703,769	28,262,463	950
95	3,058,681,425	16,691,669	1299
127	2,702,556,396	44,221,433	297

Bifrost was consistently faster than BCALM2, up to a factor 15.32, on all *k*-mer sizes and number of threads tested. For increasing *k*-mer sizes, Bifrost construction time kept decreasing while BCALM2 construction time increased. However, BCALM2 used up to 24.3% less memory than Bifrost. Memory usage for a fixed *k*-mer size was fairly constant for both tools across different number of threads, except for BCALM2 using *k*=127.

### cdBG querying

We compared Bifrost to two tools for querying dBGs based on the *k*-mer composition of the queries, namely Blight [56] and Mantis [45]. The dataset used for the graph index was the NA12878 dataset from the Genome In A Bottle consortium described in the “cdBG construction” section. For querying, Bifrost takes as input the graph it constructed and builds an index for querying *k*-mers. Mantis requires processing the unitigs of the graph with Squeakr [57] to produce a compressed table of all *k*-mers present. Mantis then builds an index directly from the output of Squeakr for querying. Blight takes as input a graph created by BCALM2. All indexes were created using *k*=31 and 16 threads.

To query the graph, we used 30 million single-end reads from the NA12878 short read dataset that was used to construct the reference graph.

Note that both Bifrost and Mantis return query hits for every query while Blight only returns the total number of *k*-mers found in the graph from all input queries. Furthermore, Mantis and Blight cannot be configured to return the presence or absence of a query based on different *k*-mer inclusion rates. Hence, Bifrost was queried initially with parameter *e*=1.0 to indicate that an input query is returned present in the graph only if all of its composing *k*-mers are present. This is done to ensure that all methods query the graph for all *k*-mers in the read. Results are shown in Table 3. Finally, Bifrost enables graph querying based on *k*-mers with up to one substitution or indel. Table 4 shows the performance of Bifrost with different *k*-mer inclusion rates, where *e*=*θ* requires at least the presence of *θ* fraction of the *k*-mers in the graph, both using exact or inexact *k*-mers. Querying for inexact *k*-mers, where an edit distance of 1 is allowed, increases the number of hits but requires more running time. However, even in the case where all *k*-mers are queried, the inexact version is still competitive with Blight and Mantis which only perform exact *k*-mer queries. Overall, the results show that Bifrost is the fastest at querying, while using 26.8 GB of memory, whereas Blight uses less memory at the expense of speed. The low memory usage of Blight is partially explained by the fact that Blight maintains its index in main memory but stores subsequences of the graph on disk.

**Table 3:** Running time and memory usage for indexing and querying a de Bruijn graph for 30 million short reads

Tool	Process	Time (m)	Memory (GB)
Bifrost	Build	**333**	39.6
	Index	**11.1**	26.8
	Query	**4.7**	26.8
	Query-total	**16.4**	26.8
BCALM2	Build	380	**35.58**
Blight	Index	80	**8.3**
	Query	13.6	**8.3**
	Query-total	93.6	**8.3**
Squeakr	Build	1147	80
Mantis	Index	54	17
	Query	38.8	168
	Query-total	96.9	168

The total time of Bifrost and Blight is split into index and query as reported by the software, whereas query-total is the wall time measurement. For Mantis, the index is a separate process and needs only to be run once

**Table 4:** Running time and fraction of queries found for different *k*-mer inclusion rates (*θ*) using exact and inexact *k*-mers

Query type	*θ*	Time (m)	Queries found (%)
Exact *k*-mers	0.50	2.8	99.0
	0.75	3.8	96.0
	0.90	4.4	93.9
	1.00	4.7	92.2
Inexact *k*-mers	0.50	7.2	99.6
	0.75	14.8	99.0
	0.90	17.7	98.1
	1.00	21.2	97.3

Inexact *k*-mers allow for one substitution or indel in the *k*-mer search

### cdBG coloring

We constructed ccdBGs with *k*=31 for a maximum of 117,913 assembled genomes of *Salmonella*. The input represents all publicly available *Salmonella* assemblies from the database Enterobase [58] as of August 2018. This is a 7.3 × increase in the number of colors compared to the work of [41] who reported the ccdBG construction for 16,000 *Salmonella* strains. We compared Bifrost to VARI-merge [41] as both tools can construct the colored de Bruijn graph and update it without reconstructing the graph entirely. The main differences between the two tools is that VARI-merge is mainly a disk-based method that produces a non-compacted colored de Bruijn graph. We only benchmarked VARI-merge as it is currently the state-of-the-art for colored de Bruijn graph construction. A comparison of VARI-merge to other colored de Bruijn graph construction tools is given in [41]. Results are given in Table 5 for a variable number of strains. Note that the reported VARI-merge time includes the time spent by KMC2 [59] to compute the *k*-mers required in input of VARI-merge.

**Table 5:** Running time, memory usage, and external disk usage for constructing the colored de Bruijn graphs of an increasing number of *Salmonella* strains

Number of strains	Tool	Time (h)	Memory (GB)	Disk (GB)
100	Bifrost	**0.016**	**0.16**	**0**
	VARI-merge + KMC2	0.33	5.1	17
400	Bifrost	**0.05**	**0.29**	**0**
	VARI-merge + KMC2	1.016	15.4	51
1600	Bifrost	**0.38**	**2.4**	**0**
	VARI-merge + KMC2	4.86	56.9	228
4000	Bifrost	**1.66**	**3.7**	**0**
	VARI-merge + KMC2	12.35	138	449
117,913	Bifrost	**93.35**	**102.74**	**0**
	VARI-merge + KMC2	N/A	N/A	N/A

N/A indicates the result is unavailable

In [41], the authors process 16,000 strains in batches of 4000, merging the batches to produce a colored de Bruijn graph of all strains. This required 254 GB of memory and 2.34 TB of external disk, with a total running time of 69 h. In comparison, Bifrost processed 117,913 strains using about 103 GB of memory, no external disk usage and a total running time of 93.35 h. While the running time is not directly comparable across different machines due to different processors, this is in line with Bifrost being about eight times faster than VARI-merge. The graph built from the 117,913 strains contains 413,658,482 *k*-mers: 39.19% of the *k*-mers have only one color (*singleton*), less than 0.01% of the *k*-mers have all the colors (*core*), and 60.80% of the *k*-mers have more than one but not all colors (*dispensable*). Among the 26,324,369 unitigs, 98.72 % have a single set of colors shared by all their *k*-mers.

## Discussion

The de Bruijn graph has been widely used as a fundamental data structure in assemblers, but the memory requirements and focus on speed mean that the implementation has been tightly integrated into the project. Bifrost allows for the integration of the de Bruijn graph as a data structure into projects that work with short read sequencing datasets or assemblies of several genomes. Reusing assemblers can often lead to suboptimal results, e.g., genome assemblers often have coverage assumptions that are not valid for transcriptome assembly. By making minimal assumptions about the input, Bifrost enables researchers to extend our work rather than having to reimplement it.

## Conclusion

We present Bifrost, a method for constructing, indexing, and querying compacted de Bruijn graphs, both regular and colored, with minimal computational requirements. Bifrost is competitive with the state-of-the-art de Bruijn graph construction method BCALM2 and the unitig indexing tool Blight with the advantage that Bifrost is dynamic. For colored de Bruijn graphs, Bifrost is about eight times faster than VARI-merge and uses about 20 times less memory with no external disk. The query capabilities of Bifrost are for both identifying colors for a given *k*-mer and navigating the de Bruijn graph. The software was developed with the intention of being usable as a tool or a library wherever large de Bruijn graphs are needed with minimal external dependencies.

## Methods

“Definitions” section details the concepts and data structures that will be used throughout this paper. “Approximating the de Bruijn graph” section describes how an approximation of the uncompacted de Bruijn graph is built from a set of sequencing reads. “Constructing the compacted de Bruijn graph” section shows how the approximate compacted de Bruijn graph is built from its uncompacted counterpart and subsequently converted to an exact compacted de Bruijn graph. “Coloring” section presents how the graph coloring is built efficiently on top of the compacted de Bruijn graph.

### Definitions

A string *s* is a sequence of symbols drawn from an alphabet $\mathcal {A}$. The length of *s* is denoted by |*s*|. A substring of *s* is a string occurring in *s*: it has a starting position *i* and a length *l* and is denoted by *s*(*i*,*l*). A substring of length *l* is also denoted an *l*-mer. In the following, we assume $\mathcal {A}$ is the DNA alphabet $\mathcal {A} = \{A, C, G, T\}$ for which symbols have complements: (*A*,*T*) and (*C*,*G*) are the complementing pairs. The reverse-complemented string $\overline {s}$ is the reverse sequence of complemented symbols in *s*. The canonical string $\hat {s}$ is the lexicographically smallest of *s* and its reverse-complement $\overline {s}$. The minimizer [60, 61] of an *l*-mer *x* is a *g*-mer *y* occurring in *x* such that *g*<*l* and *y* is the lexicographically smallest of all the *g*-mers in *x*. The lexicographical order can be cumbersome to use since poly-A *g*-mers naturally occur in sequencing data and is often replaced by a random order. The simplest way to obtain a random order is to compute a hash-value for each *g*-mer in *x* and select the *g*-mer with the smallest hash-value as the minimizer. In this work, we will only consider minimizers generated by random orderings.

**Fig. 1:**
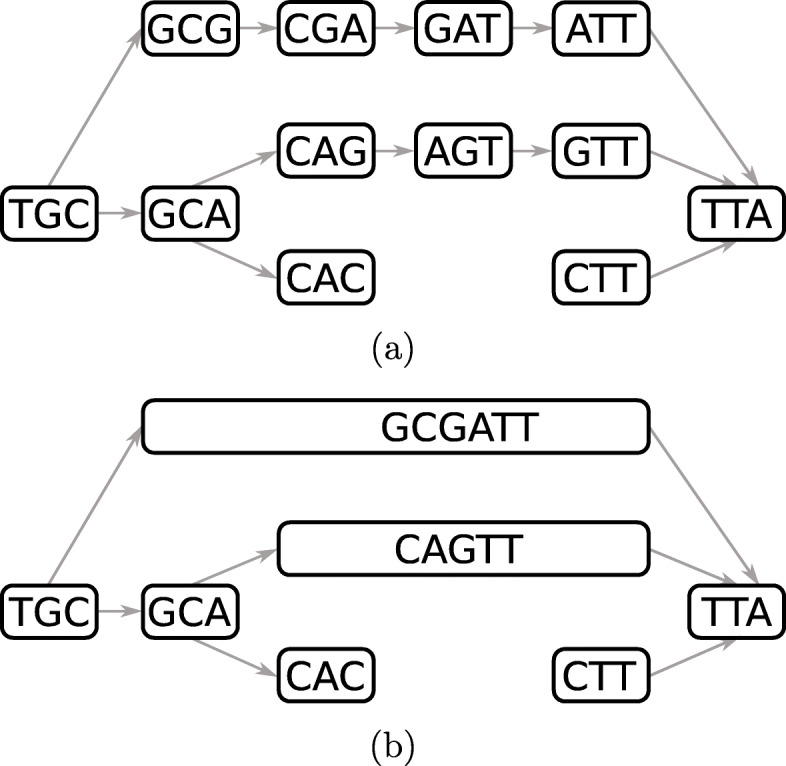
A de Bruijn graph in **a** and its compacted counterpart in **b** using 3-mers. For simplicity, reverse-complements are not considered

A de Bruijn graph (dBG) is a directed graph *G*=(*V*,*E*) in which each vertex *v*∈*V* represents a *k*-mer. A directed edge *e*∈*E* from vertex *v* to vertex *v*^′^ representing *k*-mers *x* and *x*^′^, respectively, exists if and only if *x*(2,*k*−1)=*x*^′^(1,*k*−1). Each *k*-mer *x* has $|\mathcal {A}|$ possible successors *x*(2,*k*−1)⊙*a* and $|\mathcal {A}|$ possible predecessors *a*⊙*x*(1,*k*−1) in *G* with $a\in \mathcal {A}$ and ⊙ as the concatenation operator. Note that in the original combinatorial definition of the dBG, all possible *k*-mers for an alphabet $\mathcal {A}$ are present in the graph, whereas in computational biology, the definition is restricted to a subset of the de Bruijn graph representing the *k*-mers in the input. A path in the graph is a sequence of distinct and connected vertices *p*=(*v*_1_,...,*v*_*m*_). We say that the path *p* is *non-branching* if all its vertices have an in- and out-degree of one with exception of the head vertex *v*_1_ which can have more than one incoming edge and the tail vertex *v*_*m*_ which can have more than one outgoing edge. A non-branching path is maximal if it cannot be extended in the graph without being branching. A compacted de Bruijn graph (cdBG) merges all maximal non-branching paths of *η* vertices from the dBG into single vertices, called unitigs, representing words of length *k*+*η*−1. Minimal examples of dBG and cdBG are provided in Fig. 1a and b respectively. A colored de Bruijn graph is a graph *G*=(*V*,*E*,*C*) in which (*V*,*E*) is a dBG and *C* is a set of colors such that each vertex *v*∈*V* maps to a subset of *C*; we extend the definition of a cdBG to a colored compacted de Bruijn Graph (ccdBG) to be a graph *G*=(*V*,*E*,*C*), where (*V*,*E*) is a cdBG, so the vertices represent unitigs, and each *k*-mer of a unitig maps to a subset of *C*.

Introduced by [62], the Bloom filter (BF) is a space- and time-efficient data structure that records the approximate membership of elements in a set. The BF is represented as a bitmap *B* of *m* bits initialized with 0s, coupled with a set of *f* hash functions *h*_1_,...,*h*_*f*_. Inserting and querying an element *e* into *B* is performed with the functions

$$\textsf{Insert}(e,B): B[h_{i}(e)] \gets 1 \textrm{ for all} i = 1,...,f $$

and

$$\textsf{MayContain}(e,B) : \bigwedge\limits_{i=1}^{f}B[h_{i}(e)], $$

respectively, in which $\bigwedge $ is the logical conjunction operator. Those functions require $\mathcal {O}(1)$ time. The function MayContain may report false positives when querying for elements which were never inserted but are present in *B* as a result of independent insertions. Given *n* elements to insert, the optimal number of hash functions to use [63] is $f = \frac {m}{n}\ln (2)$, for an approximate false positive rate of

$$ \varphi \approx \left(1 - e^{\frac{-fn}{m}} \right)^{f} \approx 0.7^{\frac{m}{n}} $$

Hence, the BF trades off memory usage and time complexity with a decreased false positive rate.

In order to accelerate BFs, [63] demonstrated that two hash functions combined in a double hashing technique can be applied in order to simulate more than two hash functions and obtain similar hashing performance. One main drawback of BFs is their poor data locality as bits corresponding to one element are scattered over *B*, resulting in several CPU cache misses when inserting and querying. This issue was addressed in [64], which presented the Blocked Bloom Filter (BBF), an array of smaller BFs individually fitting into one or multiple cache lines. To insert or look-up an element, a supplementary hash function is used to determine which BF to load. While BBFs are fast, their false positive ratios are usually higher than regular BFs due to the unbalanced load of each BF in the array.

As minimizers are used extensively throughout Bifrost, we use an efficient rolling hash function based on the work of [65] to select a *g*-mer as the minimizer within a single *k*-mer. Since overlapping *k*-mers are likely to share minimizers, we use an ascending minima approach [66] to recompute minimizers with amortized *O*(1) costs, so that iterating over minimizers of adjacent *k*-mers in a sequence is linear in the length of the sequence. Another optimization is to restrict the computation of minimizers to a subset of *g*-mers of a *k*-mer, namely, we exclude the first and last *g*-mer as a candidate for being a minimizer. This ensures that for a given *k*-mer, all of its forward, respectively backward, adjacent *k*-mers necessarily share the same minimizer. While it is likely that a *k*-mer *x* and its neighbor *x*^′^ share a minimizer, this neighbor hashing trick [38] guarantees that when searching all forward, respectively backward, neighbors of *x*, they will all have the same minimizer and will be stored within the same block of a BBF, thus minimizing cache misses.

### Approximating the de Bruijn graph

The *k*-mers extracted from the reads will be inserted into two BBFs: *BBF*_1_ will contain all *k*-mers occurring at least once in the input read sets while *BBF*_2_ will contain all *k*-mers occurring twice or more often. This separation allows us to filter out unique *k*-mers which are likely to be sequencing errors [67]. Algorithm 1 starts by iterating over the reads and extracts all the canonical *k*-mers. *BBF*_1_ is queried for the presence of each such *k*-mer, and *k*-mers already present in *BBF*_1_ are inserted into *BBF*_2_. Finally, *BBF*_1_ is discarded as the cdBG will be built from the *k*-mers of *BBF*_2_.


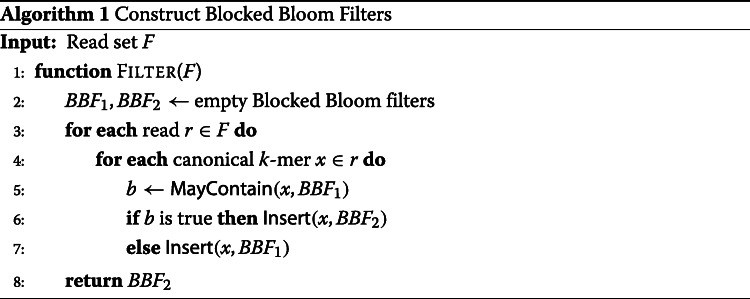


In order to accelerate the insertions into the BBFs, the minimizer hash-value of each *k*-mer is used to determine the BBF block in which the *k*-mer is inserted. This guarantees that overlapping *k*-mers sharing the same minimizer position within a read are inserted into the same BBF block, thus improving the cache efficiency of BBFs. Furthermore, the neighbor hashing of the minimizers guarantees that all predecessors and successors of a *k*-mer are hashing to the same block, thus improving graph traversal for the exact cdBG construction step. Finally, the BBFs in Bifrost use 2-choice hashing [68] to balance the number of insertions per block and reduce the number of false positives. Instead of selecting a single BBF block when inserting a *k*-mer, two blocks are selected. If none of the two blocks already contains the *k*-mer, it is inserted into the block which has the fewest number of bits set. To enable parallel insertions, each BBF block is equipped with a spinlock to avoid multiple threads inserting at the same time within the same block. Algorithm 2 refines the insertion function introduced in the “Definitions” section to enable 2-choice hashing and spinlocks usage with BBFs. Bifrost can make use of modern processors instruction sets to query simultaneously up to 16 bits within a block using AVX instructions.


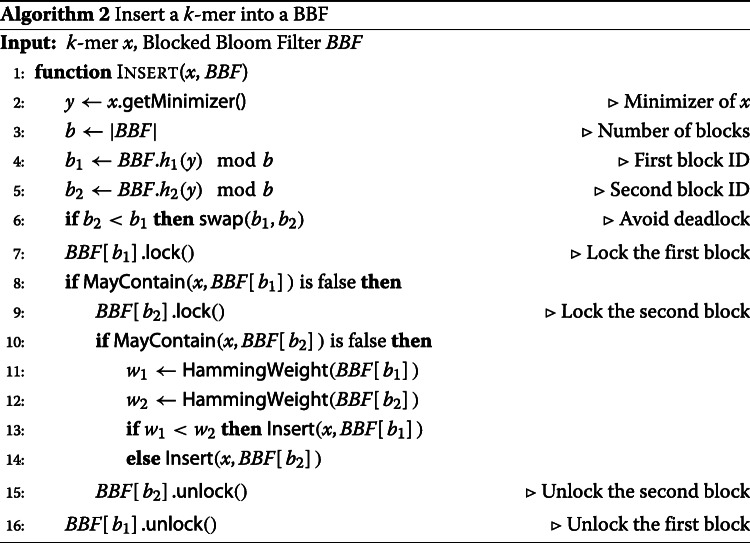


### Constructing the compacted de Bruijn graph

The following section describes the data structure indexing the unitigs. The “Unitig extraction” section details the unitig extraction procedure from the BBF and the insertion of unitigs into the cdBG data structure.

#### Data structure

**Fig. 2:**
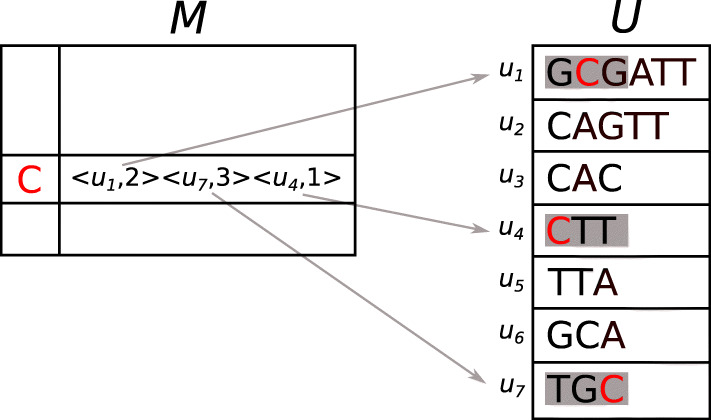
Data structure of a cdBG composed of a hash table *M* and a unitig array *U*. Unitigs are composed of 3-mers and are indexed using minimizers of length 1. For simplicity, a lexicographic ordering of minimizers is here used and only one minimizer is shown


The cdBG data structure *D*=(*U*,*M*) is composed of a unitig array *U* and a hash table of minimizers *M*. A unitig *u* is first inserted into *U* and gets a unique identifier *id*_*u*_. Unitig *u* is then decomposed into its set of constituent *k*-mers from which minimizers are extracted. Each minimizer is identified by a position *p*_*m*_ in *u*. While there can be as many minimizer positions as there are *k*-mers in the unitig, it is likely that multiple overlapping *k*-mers share the same minimizer position. The canonical *g*-mers corresponding to the minimizers are inserted into *M* and associated with their position *p*_*m*_ in *u* and the identifier *id*_*u*_. Note that a minimizer might have multiple occurrences, either within a unitig or in different unitigs of the graph. The cdBG data structure *D* is illustrated in Fig. 2. Algorithm 3 details the insertion of a unitig *u* in the cdBG data structure. Note that removing a unitig from the graph can be done in a reversed-fashion to Algorithm 3: The tuples associated with unitig *u* are removed from *M* and unitig *u* is removed from *U*.

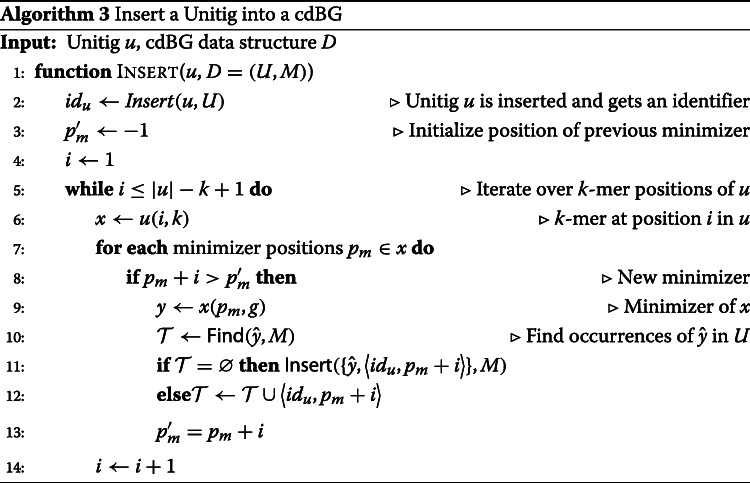


Looking-up a *k*-mer *x* in the cdBG data structure is similar to inserting a unitig. The canonical *g*-mer corresponding to the minimizer of *x* is extracted and used to query *M*. If the *g*-mer is not in *M*, *x* does not occur in a unitig of the cdBG. However, if the *g*-mer is present, the identifiers of the unitigs containing the *g*-mer and the *g*-mer positions within those unitigs are returned. *K*-mer *x* and its reverse-complement $\overline {x}$ are then anchored in those unitigs at the given minimizer positions and compared. If the comparison is positive, a tuple with the unitig identifier and the *k*-mer position in the unitig is returned. Algorithm 4 shows how to look-up *D* for a *k*-mer.


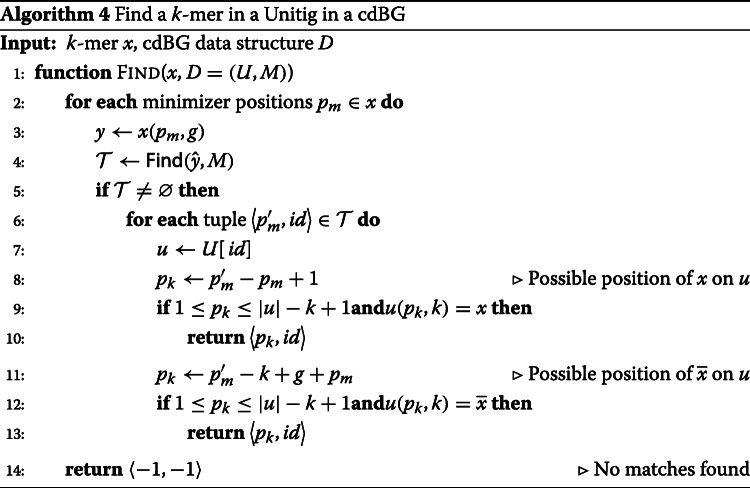


#### Unitig extraction

The BBF returned by Algorithm 1 represents an approximation of the dBG: It contains the true positive *k*-mers, namely all the *k*-mers present in the unitigs of the cdBG, but also false positive *k*-mers, which do not belong to the cdBG. The false positive *k*-mers are either artifacts of *BBF*_2_ or single occurrence *k*-mers that should have been filtered out by Algorithm 1 but were inserted into *BBF*_2_ as a result of their false occurrences in *BBF*_1_. Although BBFs are efficient data structures, they do not allow to iterate over the contents. To get around this limitation, we iterate over the original set of reads and query *BBF*_2_ to identify *k*-mers that are present.

Given a *k*-mer *x*, Algorithm 5 extracts from the BBF the unitig from which *x* is a substring, conditioned upon the presence of *x* in the BBF. *K*-mer *x* is extended forward, respectively backward, by reconstructing iteratively the prefix, respectively suffix, of the unitig using function Extend. Note that a backward extension is performed by extending forward from the reverse-complement of *x* and the extracted suffix is reverse-complemented to obtain the unitig prefix. Forward extensions are made with function ExtendForward which iteratively concatenate the last character from the next *k*-mer in the extension until no more *k*-mer is found or the extracted *k*-mer creates a cycle. Finally, *k*-mer *x* is extended with *x*^′^ using function ExtendKmer if the two *k*-mers belong to the same maximal non-branching path, i.e, if *x*^′^ is the only successor of *x* in the BBF and *x* is the only predecessor of *x*^′^ in the BBF,


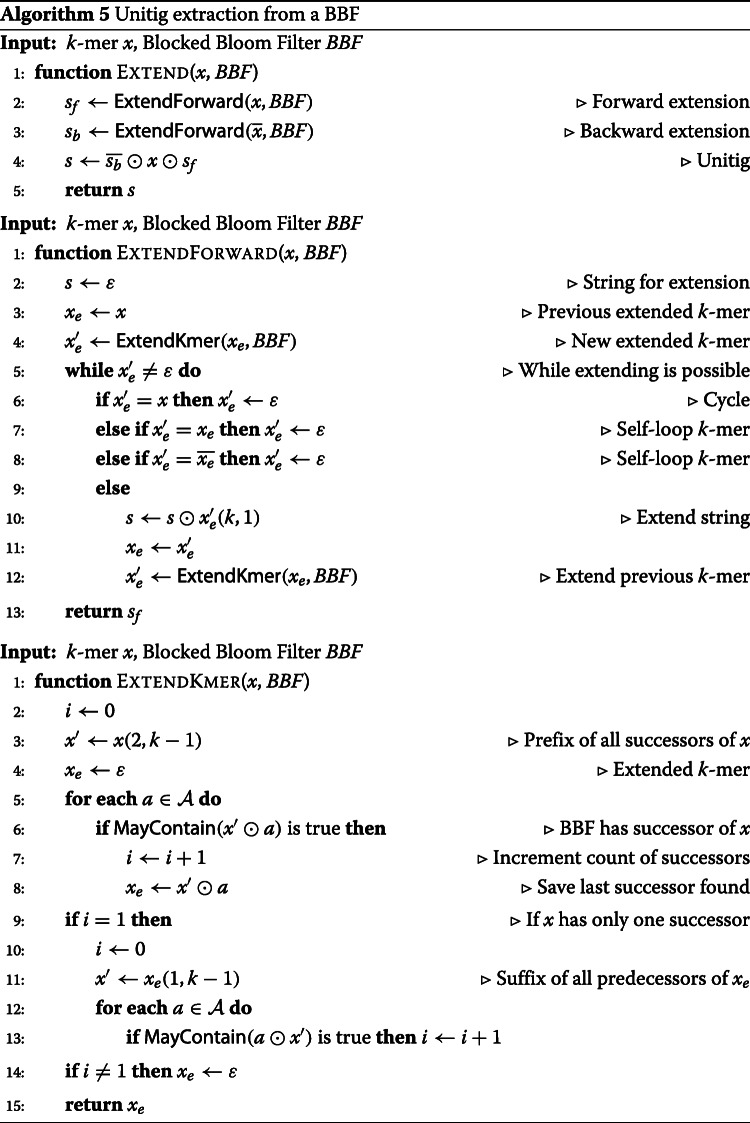


Given the read set, the BBF containing the filtered *k*-mers, and an empty cdBG data structure, Algorithm 6 extracts the unitigs from the BBF and inserts them into the cdBG data structure. The algorithm iterates over the *k*-mers of the reads and queries the BBF for their presence. A missing *k*-mer in the BBF indicates the *k*-mer was filtered out by Algorithm 1 and will not be part of a unitig, in which case the next *k*-mer in the read is queried. However, in case of the *k*-mer presence in the BBF, the cdBG is searched for the unitig containing this *k*-mer using Algorithm 4. If the *k*-mer is missing from the unitigs present in the cdBG data structure, it means its unitig has not been extracted yet from the BBF. The extraction using Algorithm 5 takes place, and the extracted unitig is inserted into the cdBG data structure with Algorithm 3.


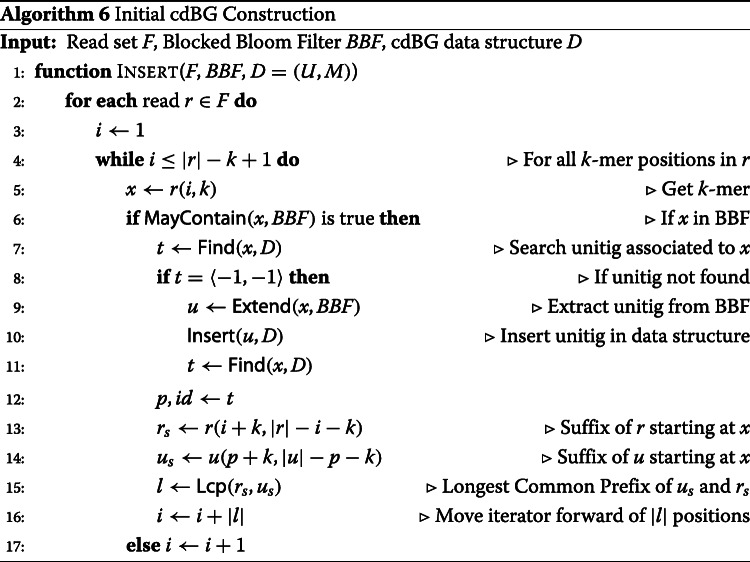


#### Eliminating the false positive *k*-mers

The cdBG constructed by Algorithm 6 is not exact as it contains false positive *k*-mers of *BBF*_2_. Those false positive *k*-mers create two types of errors in the graph:

- False connection: A false positive *k*-mer connects a unitig with no successors to a unitig with no predecessors. Hence, one unitig is extracted from the BBF instead of two.
- False branching: A false positive *k*-mer connects as a successor, respectively predecessor, to a true positive *k*-mer which already has a successor, respectively predecessor. Hence, three unitigs are extracted from the BBF instead of one.

**Fig. 3:**
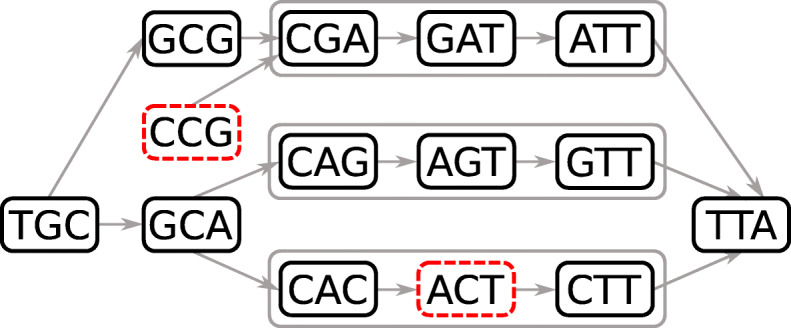
A compacted de Bruijn graph containing false positive 3-mers. Errors are represented in red dashed line vertices: *K*-mer “CCG” creates a false branching and “ACT” creates a false connection. *K*-mers that are compacted in a unitig are grouped in a gray line box

An example of a cdBG containing the two types of errors is illustrated in Fig. 3: K-mer “CCG” creates a false branching and “ACT” creates a false connection.

In order to distinguish false positive from true positive *k*-mers, a counter is maintained on each *k*-mer of the unitigs and Algorithm 6 is modified to increment the counters of the *k*-mers occurring in the reads. Hence, false positive *k*-mers with no or one single occurrence are deleted from the graph. In the case of a false connection *k*-mer, deleting the *k*-mer splits a unitig. In case of a false branching, deleting the *k*-mer joins one or multiple unitigs.


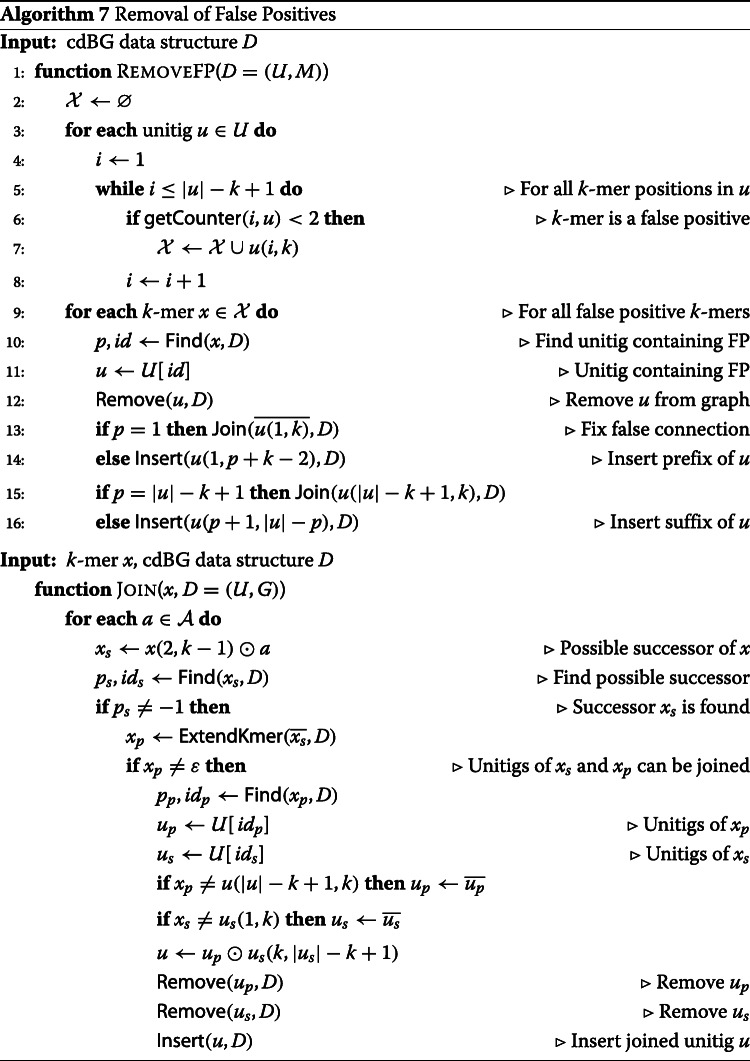


#### Ghost *k*-mers

The false positive rate of the BBF will affect the length of the unitigs extracted by Algorithm 5. Consider a unitig of length *k*+*η*−1 in the true cdBG, consisting of *ηk*-mers. For each internal *k*-mer, the algorithm makes 8 queries to the BBF, two of which will return true and 6 of which should return false. If the BBF has a false positive rate of *p*, the algorithm will advance to the next *k*-mer with probability (1−*p*)^6^≈1−6*p* and stop prematurely with probability ≈6*p*. The number of *k*-mers in the extracted unitig will then be limited by *η* on one hand and a geometric distribution with probability 6*p*, whose expected value is $\frac {1}{6p}$. When *p*=10^−3^, this would lead to an average unitig length of 167. While these errors are fixed with Algorithm 7, this leads to an increased memory usage. One way to increase the length would be to use more memory in the BBF which would reduce the false positive rate. However, we observe that the most likely configuration is that a single false positive *k*-mer *x*^′^, adjacent to a real *k*-mer *x* in the unitig, causes a premature halt to the extraction of the true unitig. When *x*^′^ has no other neighbor in the BBF except for *x*, we call it a ghost *k*-mer, insert it into a hash table to keep track of it in case we observe it later but do not stop the extraction of the unitig. In the rare case that *x*^′^ turns out to belong to the true cdBG, we identify the unitig containing *x*^′^ and fix the mistake. The probability that we halt can now be approximated as 42*p*^2^, since this would require two adjacent false positive *k*-mers to occur in the BBF. The use of ghost *k*-mers greatly reduces fragmentation which improves memory usage and running time.

#### Recurrent minimizers

Even in the case of a minimizer random ordering as described in the “Definitions” section, some minimizers are expected to occur more often in unitigs than others, due to indels occurring in homopolymer and tandem repeat sequences. Those minimizers are likely to increase the running time as their lists of tuples in the minimizer hash table *M* will be much longer than for the other minimizers. We define a minimizer as *recurrent* if it occurs *t* times or more in the unitigs of the cdBG. In order to limit the impact of recurrent minimizers on the graph construction, lists of tuples in *M* have a maximum length *t*. When a *k*-mer *x* and its corresponding minimizer *y* must be inserted into the cdBG data structure, the length of the list associated with *y* in *M* is verified first. If the length is greater or equals to *t*, *y* is a recurrent minimizer. In such case, a non-recurrent minimizer *y*^′^>*y* is extracted from *x* and inserted into *M*. If *x* does not contain a non-recurrent minimizer *y*^′^, the recurrent minimizer *y* is inserted into *M* instead. Whenever *k*-mer *x* is searched, the list of tuples associated with its minimizer *y* is traversed and *x* is anchored on the instances of *y* in the unitigs of the graph until a match is found, as described in Algorithm 4. However, if no match is found for *x* and the list of tuples associated with *y* contains *t* or more tuples, the non-recurrent minimizer *y*^′^ is extracted from *x* and the search continues using minimizer *y*^′^.

### Coloring

We denote as *D*^′^ the data structure of a ccdBG: It is composed of a unitig array *U*, a minimizer hash table *M*, an array *O* of color containers, an array *H* of hash functions, and a hash table *K* of *k*-mers.

#### Container representation

In Bifrost, a color is represented by an integer from 1 to |*C*|. A unitig *u* composed of *η*=|*u*|−*k*+1*k*-mers is associated with a binary matrix of size *η*×|*C*|: rows represent the different *k*-mer positions in *u* and columns represent the colors from *C*. A bit set at row 1≤*i*≤*η* and column 1≤*j*≤|*C*| indicates that *k*-mer *u*(*i*,*k*) occurs in dataset *j*. In order to limit the memory usage of colors, multiple compressed index is used to represent these binary matrices depending on their sparsity:

- A 64-bit word that can be either a tuple 〈position*i*,color*j*〉 or a binary matrix of size *η*×|*C*|≤62 (2 bits are reserved for the meta-data)
- A compressed bitmap adapted from a Roaring bitmap container [69]. This compressed bitmap stores up to 65488 tuples 〈position*i*,color*j*〉 and uses a maximum of 8 KB of memory. This container has 3 representations of the tuples it indexes: bit vector, sorted list of tuples, and run-length encoded list of sorted tuples. Compared to a Roaring bitmap, this compressed bitmap uses less memory for its meta-data and incurs fewer cache misses to access the tuples.
- A Roaring bitmap [69] to store more than 65488 tuples. Roaring bitmaps are SIMD accelerated and propose numerous functions to manipulate bitmaps such as set intersection and union.

Those representations have a logarithmic worst-case time look-up and insertion.


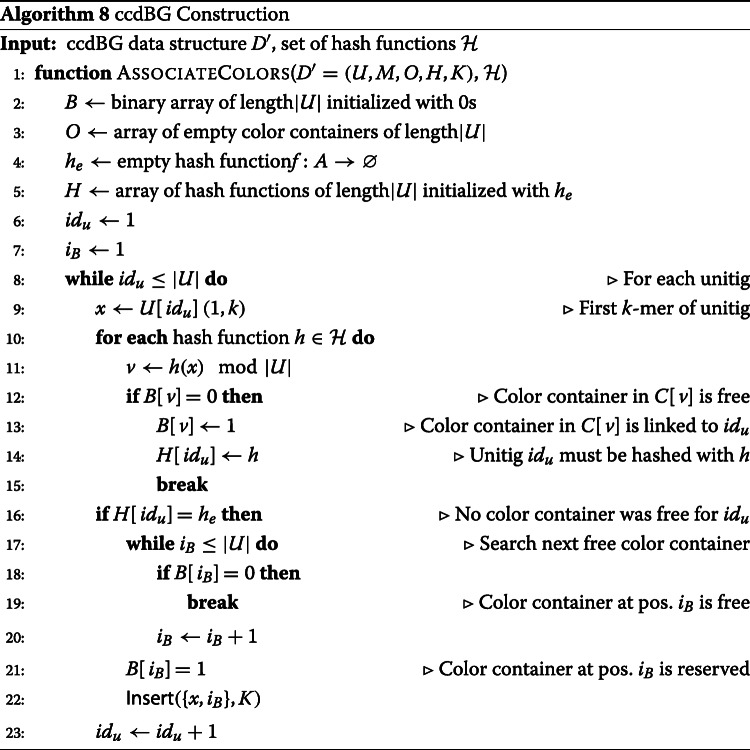


#### Container indexing

Color containers can become substantially large, and in order to avoid costly data transfer operations when the ccdBG data structure *D*^′^ is modified, color containers are not associated directly to unitigs in *D*^′^. Instead, a solution derived from the MPHF (Minimal Perfect Hash Function) library BBHash [70] is used to link unitigs of array *U* to color containers of array *O*. The benefit of such a method is that operations which affect only the structure of the graph do not move the color containers in memory. Algorithm 8 describes how color containers are associated to their respective unitigs.

## Supplementary information

**Additional file 1** Supplementary file.

**Additional file 2** Review history.

## Data Availability

We have made the source code of Bifrost available as open source software at https://github.com/pmelsted/bifrost[71]. The source code is released under a BSD-2 license. The website contains details on installation, setup, and usage. The exact version used in this paper is archived at Zenodo under https://zenodo.org/record/3973373[72].
