# Supplementary material for: Bifrost: highly parallel construction and indexing of colored and compacted de Bruijn graphs
Source: Genome Biol. 2020 Sep 17;21:249. doi: 10.1186/s13059-020-02135-8 (PMC7499882; doi:10.1186/s13059-020-02135-8)
Supplement: Supplementary file 1 — Additional file 1 Supplementary file. [file 13059_2020_2135_MOESM1_ESM.pdf]

A Availability of data and materials

We have made the source code of Bifrost available as open source software at `https://github.com/pmelsted/bifrost`. The source code is released under a BSD-2 license. The website contains details on installation, setup and usage. The exact version used in this paper is archived at Zenodo under `https://zenodo.org/record/3973373`.

Table 1 provides a list of tools used in the paper and the commit of each tool used. Table 2 lists all external datasets used.

Tool	Commit	URL
Bifrost	d252487	`https://github.com/pmelsted/bifrost`
BCALM2	9b0f4a0	`https://github.com/GATB/bcalm`
Blight	da693f6	`https://github.com/Malfoy/Blight`
Mantis	1f24ac5	`https://github.com/splatlab/mantis`
Squeakr	7d1229e	`https://github.com/splatlab/Squeakr`
VARI-Merge	b80897	`https://github.com/cosmo-team/cosmo/tree/VARI-merge`

Table 1: List of tools used

Data	URL
NA12878	`https://ftp-trace.ncbi.nlm.nih.gov/ReferenceSamples/giab/data/NA12878/NIST_NA12878_HG001_HiSeq_300x/`
Salmonella	`https://enterobase.warwick.ac.uk/species/index/senterica`

Table 2: List of datasets used

B Command lines

Command lines for each tool used in the experiments are reported here.

B.1 cdBG construction

Considering $k = 31$, 16 threads and filtering out of the construction k-mers occurring only once. BCALM2 was configured with the maximum memory usage of Bifrost for each k-mer size tested.

- Bifrost

```
$ Bifrost build -v -t 16 -k 31 -s GIAB-samples.txt -o GIAB-bifrost-k31
```

- BCALM2

```
$ bcalm -nb-cores 16 -kmer-size 31 -abundance-min 2 -max-memory 40556
-in GIAB-samples.txt -out GIAB-bcalm-k31
```

B.2 cdBG querying

- Bifrost

```
$ Bifrost query -v -e 1.0 -t 16 -k 31 -g GIAB-bifrost-k31.gfa -q
queries.fastq -o out
```

- Blight

```
$ bench_blight -t 16 -k 31 -g GIAB-bcalm-k31.unitigs.fa -q
queries.fastq
```

- Squeakr and Mantis

```
$ squeakr count -e -s 34 -k 31 -n -c 2 -t 16 -o sq31/output
R1.fastq.gz R2.fastq.gz
$ mantis build -s 32 -i sq31/output -o mantis
$ mantis query -1 -p mantis/ -o output queries.fastq
```

B.3 cdBG coloring

- Bifrost

```
$ Bifrost build -v -t 16 -k 31 -c -r salmonella-genomes.txt -o
salmonella-bifrost-k31
```

- VARI-merge

```
$ ls -1 *.fasta | xargs -l -i cosmo/3rd_party_src/KMC/bin/kmc -ci0 -fm
-k31 -cs300  _kmc kmc_temp
$ ls -1 *.fasta | xargs -l -i cosmo/3rd_party_src/KMC/bin/kmc_tools
sort _kmc _kmc_sorted_kmc.kmc
$ ls -1 *.fasta | xargs -l -i echo "_kmc_sorted_kmc.kmc" > files.list
$ cosmo-build -d files.list
$ pack-color files.list.colors 10 128970820 94946816
```
