# Supplementary material for: Bifrost: highly parallel construction and indexing of colored and compacted de Bruijn graphs
Source: Genome Biol. 2020 Sep 17;21:249. doi: 10.1186/s13059-020-02135-8 (PMC7499882; doi:10.1186/s13059-020-02135-8)
Supplement: Supplementary file 2 — Additional file 2 Review history. [file 13059_2020_2135_MOESM2_ESM.docx]

Review History

**First round of review**

**Reviewer 1**

**Were you able to assess all statistics in the manuscript, including the appropriateness of statistical tests used?**

There are no statistics in the manuscript.

**Were you able to directly test the software?**

Yes

**Comments to author:**

The authors present an algorithm for building a compacted de Bruijn graph (dBG) (i.e., unary paths are collapsed into single units) directly from a set of DNA sequences without producing intermediate uncompacted representations. They also propose a method for encoding colors if the dBG is colored (augmented with extra data).

Briefly, the whole idea can be described as follow:

They scan an input DNA collection (say $R$) and store its kmers in a Blocked Bloom Filter (BBF), an efficient (and probabilistic) data structure that records the membership of elements in a set.

They query the kmers of $R$ in the BFF and expand them forward and backward to produce the unitigs of the dBG. The unitigs are then saved into a data structure $D$, composed of an array $U$ that keeps the sequences, and a hash table $M$, that records the different minimizers in $U$.

As the BFF is a probabilistic data structure, it may produce false-positive kmers and, hence, erroneous unitigs. Thus, a final step that removes incorrect sequences is also carried out.

Major comments:

Overall, I think it is reasonable work. Experimental results show the proposed algorithms perform better than other similar techniques (BCALM2) in both working space and running time. The code in GitHub seems to be well developed.

Still, some critical aspects are not well developed, and for that reason, I cannot accept the article as it is. I list them below:

There is no empirical evaluation of how the value of $k$ affects the performance of the proposed algorithms. The number we choose for $k$ changes the size and the topology of the graph. I would expect this to have an impact on your tool.

They do not report the space used by $D$ or the time required for querying the compacted graph (outdegree, indegree, successor, predecessor, etc.). I stress this because I have the impression that $D$ uses more space than the ~ |E|\log\sigma + o(|E|\log \sigma) bits of BOSS, the BWT-based dBG representation used by VARI. $U$ uses at least $|E|\log sigma$ bits, and the space of $M$ can be significant depending on the implementation of the hash table. Having an efficient algorithm for building a compact dBG is relevant, of course. But if the resulting representation ($D$) performs worse than other existing succinct data structures (BOSS), then it becomes less relevant for the final users.

Regarding the previous point, I recommend storing the unitigs using a BWT-based index instead of $D$. Most of the algorithms remain the same, except maybe Algorithm 8.

The quality of the final unitigs obtained by their method is not assessed. I would recommend comparing unitig sizes versus different false-positive rates or kmer sizes. I would also use other dBG representations for making some other comparisons. For instance, (i) I would simulate some reads from a well-assembled genome, then (ii) I would produce unitigs with your approach, but also with BOSS, and then (iii) I would compare sizes, synteny, etc.

Minor comments:

What kind of hashing are you using for $M$? I mention this because it would be more efficient to use perfect hashing if the number of minimizers is fixed.

It would be interesting to show the space used by the BFF. I will also show the fraction of space used by every element of $D$ ($U$ and $M$).

The time for retrieving the colors of an edge is not evaluated. By the way, why coloring every kmer inside a unitig? Isn't a unitig supposed to appear in all the subsets? Maybe I misunderstood the idea (Section 3.3.1)

Section 3.2.2 (first 3 lines), I think the description of what an approximation of the dBG means is not well written.

Algorithm 3, line 10, what does $\^{y}$ mean?

Algorithm 5, line 7, what does $X_f$ mean?

Algorithm 7, function RemoveFP, line 9, did you mean $\mathcal{X}$?

Algorithm 7, function Join, lines 4 and 8, you forgot verbatim font for function Find.

3.3.1 Container representation, "requires less cache-miss to access the tuples," -> incurs fewer cache-misses to access the tuples.

**Reviewer 2**

**Were you able to assess all statistics in the manuscript, including the appropriateness of statistical tests used?**

There are no statistics in the manuscript.

**Were you able to directly test the software?**

Yes

**Comments to author:**

In this manuscript, Holley and Melsted described Bifrost, a software package to construct and index colored compacted de Bruijn graph (ccdBG). There have been several related tools for this task (see below). The authors demonstrated that Bifrost is either more efficient or more versatile than the existing tools. I have compiled and installed Bifrost. It is reasonably easy to install. The manuscript is generally well written. I don't have detailed comments on the algorithm part. Critical observations focus on the introduction and the results part.

1) Biological applications are preferred. The current manuscript is good for a paper in computer science. However, for a Genome Biology paper, I am expecting something more related to biology. In Section 4.1, the authors assembled short reads. However, such an assembly is barely useful for downstream applications. In practice, we correct sequencing errors (e.g. as in SGA and SPAdes), use relatively long k-mers (e.g. ALLPATHS-LG uses >90bp; many assemblers use 50bp-ish) and apply sophisticated graph cleaning techniques in addition to tip trimming. I wonder how hard it is to combine kallisto and bifrost. This would make a very powerful tool. In Section 4.2, the authors only evaluate the performance of Bifrost on pan-genome graph construction. I think the natural next step is to analyze this graph: the number of vertices/edges, the average/N50 vertex length, the size of the "core genome", etc. Having this would dramatically improve the biological part of this manuscript. Some basic statistics is ok.

2) It would be good to have a more systematic review of the field. There are multiple tools related to ccdBG. However, their purposes are subtly different. For example, some focus on reads; some focus on multiple genomes; some keep colors; some index existing graphs. I honestly don't know their exact differences. I guess many readers will have the same question: how is bifrost (un)related to tools like splitmem, twopaco, bcalm2/blight, rainbowfish/pufferfish/mantis+mst, VARI/VARI-merge, etc?

3) I wonder how hard it is to add more tools to the evaluation. I think twopaco and something from the rainbowfish/pufferfish/mantis+mst series should be evaluated. There are only several related tools in this area.

4) Evaluating the query performance is needed. The title of this manuscript mentions "indexing". Table 1 includes Blight, which is a graph indexed. The role of indexing is to perform query. However, no query performance is given. As a matter of fact, the Bifrost executable doesn't have a query command. Bifrost creates an index which is currently not of much use to end users. The authors many learn from the pufferfish paper about how to evaluate the query performance.

Minor comments:

5) In my opinion, the authors should emphasize the versatility of bifrost (maybe with a table). My understanding is that bifrost works for both short reads and pangenomes, keeps colors and indexes graphs as well. Perhaps mantis+mst is the closest in terms of features?

6) Bifrost uses two differen command line options for full genomes vs short reads. What is the difference between the two? Similarly, bcalm2 emphasizes on short reads, while twopaco talks more about pangenomes. Is there a specific reason for this? I can actually run bcalm2 for multiple full genomes with correct output.

7) In my view, it is worth giving deBGA a highlight. deBGA is a complete short-read mapper. It relates unitigs to the input genomes and goes further than most tools relevant to cdBG (though perhaps this is not a feature hard to implement).

8) I personally have concerns with the command-line syntax of Bifrost. In particular, bifrost asks users to provide multiple files with spaces, following -s/-r. This is the not unix way and may confuse users. The right way is to accept multiple -s/-r and/or use comma-separated list.

9) It seems that bcalm2 by default supports k-mers longer than 32bp. However, bifrost has to trade memory for longer k-mers. In Table 1, what if the authors use 51-mer, which is more common in human assembly?

Reviewer: Heng Li

**Reviewer 3**

**Were you able to assess all statistics in the manuscript, including the appropriateness of statistical tests used?**

Yes, and I have assessed the statistics in my report.

**Were you able to directly test the software?**

Yes

**Comments to author:**

The authors present a novel tool for construction of colored condensed de Bruijn graphs just in memory using lots of clever algorithmic and programming techniques. While being very interesting we're seeing number of points that could be addressed / improved as well as the issues that we believe must be fixed to enable broad usage. We have split these issues into several categories

- Source code itself

1. One the key build-time parameters is maximum k-mer length. Can it be made CMAKE option? So one would not need to hack the sources or do some weird things like adding define to CFLAGS, etc.

2. The authors provides bfrost also as library. Can the cmake scripts be modified, so it could be easily integrated into existing project? Ideally one should just include the necessary CMakeLists.txt file, and then target_link_library() would do all the necessary lifting including setting the paths to the headers, etc.

3. The maximum k-mer length is not only the build-time parameter, but a part of library ABI. Some additional precautions should be made to ensure that the library built with one max k-mer size setting are not linked with the objects compiled with the other one (especially important when bfrost is built as a dynamic library). Can some name mangling be done here to ensure that max k-mer size is a part of symbol names, etc.?

- Usage

1. It's a bit strange that setting MAX_KMER_SIZE to, say, 128 effectively disallows k=128. What is the reason? So to be on par with different tools and try k-mer of 128 (or 127 if we're using a bit different graph terminology) we'd need to set MAX_KMER_SIZE being larger than 128.

2. Something is really strange wrt the performance. I tried to compare the results with spades-gbuilder (which is basically de Bruijn graph condenser from SPAdes package). I took just two reference genomes: E. coli str. K12 and E. coli DH10B and did the following experiments:

2.1 Built graphs in reference mode with Bfrost (using -k 56) and spades-gbuilder (-k 55). The results seems to be identical, at least the number of edges is the same. However, the overall timing seems to be odd to me:

- spades-gbuilder in default mode finished in 4.41 seconds

- Bfrost -t 1 finished in 6.67 wall seconds (and 6.60 user time)

- Bfrost -t 16 finished in 6.9 wall second (and 16.60 user time)

So, where is speedup?

2.2 The situation seems to be much worse with -k 126 (I cannot use -k 128 to be on par with spades-gbuilder / BCAML)

- spades-gbuilder finished in 7 seconds

- Bfrost -t 16 -k 126 took 53 seconds and generated output file of 121 Mbytes which does not seem right.

Can this problem be fixed? It seems quite serious to me...

- Benchmarking and presentation of results

1. Can particular source code revisions and command lines of tools to be included for the sake of reproducibility?

2. How does Bfrost scale? Can we see some additional graphs? E.g. dependency of memory consumption and construction speed on # of threads, k-mer lengths, etc.?

**Authors Response**

**Point-by-point responses to the reviewers’ comments:**

*We thank the reviewers for their constructive comments, which we believe have improved both the paper and the software. We provide a point-by-point response to the comments raised by the reviewers below.*

Reviewer #1:

The authors present an algorithm for building a compacted de Bruijn graph (dBG) (i.e., unary paths are collapsed into single units) directly from a set of DNA sequences without producing intermediate uncompacted representations. They also propose a method for encoding colors if the dBG is colored (augmented with extra data).

Briefly, the whole idea can be described as follow:

They scan an input DNA collection (say $R$) and store its kmers in a Blocked Bloom Filter (BBF), an efficient (and probabilistic) data structure that records the membership of elements in a set.

They query the kmers of $R$ in the BFF and expand them forward and backward to produce the unitigs of the dBG. The unitigs are then saved into a data structure $D$, composed of an array $U$ that keeps the sequences, and a hash table $M$, that records the different minimizers in $U$.

As the BFF is a probabilistic data structure, it may produce false-positive kmers and, hence, erroneous unitigs. Thus, a final step that removes incorrect sequences is also carried out.

Major comments:

Overall, I think it is reasonable work. Experimental results show the proposed algorithms perform better than other similar techniques (BCALM2) in both working space and running time. The code in GitHub seems to be well developed.

Still, some critical aspects are not well developed, and for that reason, I cannot accept the article as it is. I list them below:

There is no empirical evaluation of how the value of $k$ affects the performance of the proposed algorithms. The number we choose for $k$ changes the size and the topology of the graph. I would expect this to have an impact on your tool.

*A comparison of Bifrost to BCALM2 using four k-mer sizes (31, 63, 95 and 127) and four numbers of threads (1, 4, 8 and 16) is now provided in the paper in Table 1.*

They do not report the space used by $D$ or the time required for querying the compacted graph (outdegree, indegree, successor, predecessor, etc.). I stress this because I have the impression that $D$ uses more space than the ~ |E|\log\sigma + o(|E|\log \sigma) bits of BOSS, the BWT-based dBG representation used by VARI. $U$ uses at least $|E|\log sigma$ bits, and the space of $M$ can be significant depending on the implementation of the hash table. Having an efficient algorithm for building a compact dBG is relevant, of course. But if the resulting representation ($D$) performs worse than other existing succinct data structures (BOSS), then it becomes less relevant for the final users.

*$D$ is a fully dynamic data structure while the BOSS representation only provides merging as dynamic operation. Hence, comparing both data structures solely on their memory is not possible. While we agree that $D$ uses theoretically more space than the BOSS representation, VARI-Merge is based on the BOSS representation and our experiments demonstrate that Bifrost outperforms VARI-Merge in terms of memory usage (see Table 1)*

Regarding the previous point, I recommend storing the unitigs using a BWT-based index instead of $D$. Most of the algorithms remain the same, except maybe Algorithm 8.

*As previously stated, Bifrost uses a fully dynamic data structure while most BWT-based data structures only provide merging as dynamic operation. Furthermore the dynamic index is used throughout the construction of the cdBG, where it is essential that we can modify the unitigs, by splitting or joining them as we fix the graph. Hence, replacing it with a BWT-based data structure is not an option at this point. Since the output of the build command is a graph in GFA format, other software could be developed that indexes graphs based on BWT (unitigs only) or extensions of BWT to graphs, but this is beyond the scope of this paper.*

The quality of the final unitigs obtained by their method is not assessed. I would recommend comparing unitig sizes versus different false-positive rates or kmer sizes. I would also use other dBG representations for making some other comparisons. For instance, (i) I would simulate some reads from a well-assembled genome, then (ii) I would produce unitigs with your approach, but also with BOSS, and then (iii) I would compare sizes, synteny, etc.

*Bifrost builds an exact and deterministic compacted de Bruijn graph. Hence, the false positive rate of the Bloom filter in Bifrost has no impact on its output, only the memory usage and running time are affected. In our experiments, unitigs produced by Bifrost were the same as the ones produced by BCALM2. It was not possible to compare to the unitigs of VARI-Merge, which is based on the BOSS representation, as the output is a binary format while Bifrost and BCALM2 output GFA, a widely used and community supported file format for assembly graphs.*

Minor comments:

What kind of hashing are you using for $M$? I mention this because it would be more efficient to use perfect hashing if the number of minimizers is fixed.

*Bifrost uses two different hash functions: the rolling hash function RepHash (Melsted and Halldórsson, 2011) and xxHash (Yann Collet, https://github.com/Cyan4973/xxHash ) for a stronger hashing (passes the SMHasher test suite).*

*While perfect hashing is not used directly to index minimizers or unitigs, Section 4.3.2 describes how we use a Minimal Perfect Hash Function derived from BBHash (Limasset et al., 2017) to index the color containers of the graph.*

It would be interesting to show the space used by the BFF. I will also show the fraction of space used by every element of $D$ ($U$ and $M$).

*The space usage of the BFF is a function of the number of distinct k-mers and will vary by dataset. Regarding the space usage of elements of $D$, we believe that reporting this number would not lead to insights for the reader and thus do not report it in the interest of readability.*

*The time for retrieving the colors of an edge is not evaluated. By the way, why coloring every kmer inside a unitig? Isn't a unitig supposed to appear in all the subsets? Maybe I misunderstood the idea (Section 3.3.1)*

Colors are not associated with edges but instead, to k-mers within unitigs.

*A unitig is a compacted maximal non-branching path of the de Bruijn graph built from all the k-mers of all the dataset. As such, unitigs are not required to appear in one, some or all dataset. A simple example is to build the ccdBG from two dataset 1 and 2. Dataset 1 yields a single unitig A while dataset 2 yields a single unitig B. When building the ccdBG from dataset 1 and 2, it is possible that the only k-mer overlap is the last k-mer of A overlapping the first k-mer of B. Hence, A and B would merge into a single unitig C. Yet, C does not occur in dataset 1 nor dataset 2. However, by coloring individually the k-mers of unitig C, it is possible to identify which segments of C occur respectively in dataset 1 and 2. This representation differs from the one of SplitMEM in which unitigs are split where color classes from overlapping k-mers diverge. However, SplitMEM does not represent exactly a compacted de Bruijn graph since nodes are not exactly unitigs.*

*In most cases the k-mers of the unitigs have the same color and this is used extensively in the compression scheme of the color containers.*

Section 3.2.2 (first 3 lines), I think the description of what an approximation of the dBG means is not well written.

Those 3 lines read:

"The BBF returned by Algorithm 1 represents an approximation of the dBG: It contains the true positive k-mers composing the unitigs of the cdBG and false positive k-mers to exclude from the cdBG construction."

*We have modified this to read:*

*'The BBF returned by Algorithm 1 represents an approximation of the dBG: It contains the true positive $k$-mers, namely all the $k$-mers present in the unitigs of the cdBG, but also false positive $k$-mers, which do not belong to the cdBG.'*

Algorithm 3, line 10, what does $\^{y}$ mean?

*$\^{y}$ is the canonical minimizer of $y$ (see definitions in Section 2).*

Algorithm 5, line 7, what does $X_f$ mean?

*We thank the reviewer for noticing this typo. It should have been $X_e$ and it has been changed in the paper.*

Algorithm 7, function RemoveFP, line 9, did you mean $\mathcal{X}$?

*Indeed, the math notation $\mathcal{}$ had been omitted on $X$ and it has been changed in the paper.*

Algorithm 7, function Join, lines 4 and 8, you forgot verbatim font for function Find.

*Indeed, the verbatim notation had been omitted on function Find() in Algorithm 7 and it has been changed in the paper.*

3.3.1 Container representation, 'requires less cache-miss to access the tuples,' -> incurs fewer cache-misses to access the tuples.

*This was changed in the paper.*

Reviewer #2:

In this manuscript, Holley and Melsted described Bifrost, a software package to construct and index colored compacted de Bruijn graph (ccdBG). There have been several related tools for this task (see below). The authors demonstrated that Bifrost is either more efficient or more versatile than the existing tools. I have compiled and installed Bifrost. It is reasonably easy to install. The manuscript is generally well written. I don't have detailed comments on the algorithm part. Critical observations focus on the introduction and the results part.

1)	Biological applications are preferred. The current manuscript is good for a paper in computer science. However, for a Genome Biology paper, I am expecting something more related to biology. In Section 4.1, the authors assembled short reads. However, such an assembly is barely useful for downstream applications. In practice, we correct sequencing errors (e.g. as in SGA and SPAdes), use relatively long k-mers (e.g. ALLPATHS-LG uses >90bp; many assemblers use 50bp-ish) and apply sophisticated graph cleaning techniques in addition to tip trimming. I wonder how hard it is to combine kallisto and bifrost. This would make a very powerful tool. In Section 4.2, the authors only evaluate the performance of Bifrost on pan-genome graph construction. I think the natural next step is to analyze this graph: the number of vertices/edges, the average/N50 vertex length, the size of the "core genome", etc. Having this would dramatically improve the biological part of this manuscript. Some basic statistics is ok.

*Two biological applications of Bifrost have been added to the manuscript. Bifrost is the core data structure in the SANS software for alignment- and reference-free phylogenomics (Wittler, Proceedings of WABI, 2019) as well as the tool BlastFrost for bacterial genomes querying of genes linked to pathogenicity islands and fluoroquinolone resistance (Luhmann et al, biorxiv, 2020). Six other projects, without paper nor preprint at the moment, are under active development using Bifrost.*

*We clarified our Results section by mentioning that Bifrost was benchmarked in three representative use-cases: construction, querying and coloring. Our intention is to show that Bifrost is suitable for assembly-related problems but we do not claim that Bifrost is a genome assembler nor does it perform assembly.*

*Compared to the first submitted manuscript, Bifrost has been benchmark for sequence querying. The experiments are reported in the corresponding section of the paper.*

*The coloring section has been augmented with statistics about the graph built and core / accessory / singleton k-mers.*

2)	It would be good to have a more systematic review of the field. There are multiple tools related to ccdBG. However, their purposes are subtly different. For example, some focus on reads; some focus on multiple genomes; some keep colors; some index existing graphs. I honestly don't know their exact differences. I guess many readers will have the same question: how is bifrost (un)related to tools like splitmem, twopaco, bcalm2/blight, rainbowfish/pufferfish/mantis+mst, VARI/VARI-merge, etc?

*The paper now contains a section “Previous work” focusing solely on tools for constructing the compacted de Bruijn graph, with and without colors. Three survey paper references are also provided to give a more detailed overview of tools using de Bruijn graphs, compacted or not, for different applications.*

3)	I wonder how hard it is to add more tools to the evaluation. I think twopaco and something from the rainbowfish/pufferfish/mantis+mst series should be evaluated. There are only several related tools in this area.

*BCALM2 is the state-of-the-art when it comes to compacted de Bruijn graph construction which is why it is evaluated against Bifrost in the assembly section. TwoPaCo was discarded from the comparison as it can only build the graph from fully assembled genomes.*

*Pufferfish indexes the compacted de Bruijn graph but does not build it. It was therefore discarded from the graph construction benchmark. Mantis is benchmarked in the querying section.*

4)	Evaluating the query performance is needed. The title of this manuscript mentions "indexing". Table 1 includes Blight, which is a graph indexed. The role of indexing is to perform query. However, no query performance is given. As a matter of fact, the Bifrost executable doesn't have a query command. Bifrost creates an index which is currently not of much use to end users. The authors many learn from the pufferfish paper about how to evaluate the query performance.

*A “query” command has been added to the Bifrost CLI, allowing to query the graph for exact and inexact k-mers using a threshold of the k-mer quorum that can be changed. The sequence querying can be for the entire graph or per color in the graph. This command has been benchmarked against Blight and Mantis in the Results section.*

Minor comments:

5)	In my opinion, the authors should emphasize the versatility of bifrost (maybe with a table). My understanding is that bifrost works for both short reads and pangenomes, keeps colors and indexes graphs as well. Perhaps mantis+mst is the closest in terms of features?

*We have incorporated this suggestion in the Introduction and Conclusion.*

6)	Bifrost uses two differen command line options for full genomes vs short reads. What is the difference between the two? Similarly, bcalm2 emphasizes on short reads, while twopaco talks more about pangenomes. Is there a specific reason for this? I can actually run bcalm2 for multiple full genomes with correct output.

*Bifrost CLI for full genomes builds the graph from k-mers occurring once or more while the CLI for short reads builds the graph from k-mers occurring twice or more. It is similar to using --min-abundance 1 and --min-abundance 2 in BCALM2. BCALM2 can run on full genomes (--min-abundance 1) or short reads (--min-abundance 2+). However, TwoPaCo can only be built from full genomes. Both BCALM2 and TwoPaCo do not index colors nor, more generally, sample identity.*

7)	In my view, it is worth giving deBGA a highlight. deBGA is a complete short-read mapper. It relates unitigs to the input genomes and goes further than most tools relevant to cdBG (though perhaps this is not a feature hard to implement).

*deBGA was cited in the Introduction of the submitted manuscript. Section “Previous work” describes deGSM, a compacted de Bruijn graph construction tool by some of the authors of deBGA. As it is difficult to describe deBGA without giving a highlight to the many tools revolving around compacted de Bruijn graphs, section “Previous work” focuses solely on tools for constructing the compacted de Bruijn graphs, with and without colors, in the interest of keeping the manuscript concise.*

8)	I personally have concerns with the command-line syntax of Bifrost. In particular, bifrost asks users to provide multiple files with spaces, following -s/-r. This is the not unix way and may confuse users. The right way is to accept multiple -s/-r and/or use comma-separated list.

*Bifrost CLI has been modified accordingly.*

9)	It seems that bcalm2 by default supports k-mers longer than 32bp. However, bifrost has to trade memory for longer k-mers. In Table 1, what if the authors use 51-mer, which is more common in human assembly?

*A comparison of Bifrost to BCALM2 using four k-mer sizes (31, 63, 95 and 127) and four numbers of threads (1, 4, 8 and 16) is now provided in the paper (Table 1)*

Reviewer: Heng Li <[hli@jimmy.harvard.edu](mailto:hli@jimmy.harvard.edu)>

Reviewer #3: The authors present a novel tool for construction of colored condensed de Bruijn graphs just in memory using lots of clever algorithmic and programming techniques. While being very interesting we're seeing number of points that could be addressed / improved as well as the issues that we believe must be fixed to enable broad usage. We have split these issues into several categories

- Source code itself

1. One the key build-time parameters is maximum k-mer length. Can it be made CMAKE option? So one would not need to hack the sources or do some weird things like adding define to CFLAGS, etc.

*The README file on the GitHub repository has been modified to indicate how to compile Bifrost for larger k-mer sizes using the option -DMAX_KMER_SIZE when calling cmake.*

2.	The authors provides bfrost also as library. Can the cmake scripts be modified, so it could be easily integrated into existing project? Ideally one should just include the necessary CMakeLists.txt file, and then target_link_library() would do all the necessary lifting including setting the paths to the headers, etc.

*We believe that the cmake file of Bifrost is simple enough to be modified easily and integrated into other projects, as the the tool BlastFrost ( https://github.com/nluhmann/BlastFrost ) shows . A pull request to make Bifrost integration even more easy is currently in progress on the GitHub repository of Bifrost and we hope to have it accepted soon after further testing.*

3.	The maximum k-mer length is not only the build-time parameter, but a part of library ABI. Some additional precautions should be made to ensure that the library built with one max k-mer size setting are not linked with the objects compiled with the other one (especially important when bfrost is built as a dynamic library). Can some name mangling be done here to ensure that max k-mer size is a part of symbol names, etc.?

*We believe it is indeed possible but will require some further software engineering to get it done. The difficulty is not much to have this name mangling for the binary but to have it integrated transparently in the API such that it does not become a burden for our users.*

- Usage

1.	It's a bit strange that setting MAX_KMER_SIZE to, say, 128 effectively disallows k=128. What is the reason? So to be on par with different tools and try k-mer of 128 (or 127 if we're using a bit different graph terminology) we'd need to set MAX_KMER_SIZE being larger than 128.

*It makes sense and we would like to thank the authors for this suggestion. This change has been integrated into Bifrost’s roadmap and will be released soon.*

2.	Something is really strange wrt the performance. I tried to compare the results with

spades-gbuilder (which is basically de Bruijn graph condenser from SPAdes package). I took

just two reference genomes: E. coli str. K12 and E. coli DH10B and did the following

experiments:

2.1 Built graphs in reference mode with Bfrost (using -k 56) and spades-gbuilder (-k 55). The

results seems to be identical, at least the number of edges is the same. However, the overall

timing seems to be odd to me:

- spades-gbuilder in default mode finished in 4.41 seconds

- Bfrost -t 1 finished in 6.67 wall seconds (and 6.60 user time)

- Bfrost -t 16 finished in 6.9 wall second (and 16.60 user time)

So, where is speedup?

*Micro-benchmarks on very small input can be a misleading way of measuring software efficiency. In this example, the input data is so small that both tools very likely spend more time on IO from disk and initializing internal data structures rather than actually computing the de Bruijn graph. This also applies to the comparison of Bifrost single thread to multi-threaded: compute (wall-clock) time is mostly the same because the tool spends more time initializing threads rather than threads performing the computation.*

*In general, I would advise providing both tools enough data to compute for about at least 30s. This will give enough time to both tools to initialize internal structures such as threads and making sure that the impact of external factors is minimized on the running time of both tools. The running time will then reflect the true time efficiency of both tools.*

.

2.2 The situation seems to be much worse with -k 126 (I cannot use -k 128 to be on par with spades-gbuilder / BCAML)

- spades-gbuilder finished in 7 seconds

- Bfrost -t 16 -k 126 took 53 seconds and generated output file of 121 Mbytes which does not seem right.

Can this problem be fixed? It seems quite serious to me...

*We would like to thank the reviewer for noticing this issue. It has been solved, tested and a comparison of Bifrost to BCALM2 with k=127 is provided in the paper using different numbers of threads.*

- Benchmarking and presentation of results

1.	Can particular source code revisions and command lines of tools to be included for the sake of reproducibility?

*All source code versions and revisions as well as command lines have been put in the Appendix.*

2.	How does Bfrost scale? Can we see some additional graphs? E.g. dependency of memory consumption and construction speed on # of threads, k-mer lengths, etc.?

*A comparison of Bifrost to BCALM2 using four k-mer sizes (31, 63, 95 and 127) and four numbers of threads (1,4,8 and 16) is now provided in the paper.*

**Second round of review**

**Reviewer 2**

The authors have addressed my major concerns. I only have one minor comment.

1) In response to Reviewer 1, the authors showed an example to explain why k-mers on one unitig may be associated with different sets of colors. I was aware of this example. As the authors now show the statistics on the pangenome graph (which I appreciate), I have a follow-up question. We define a unitig to be "color consistent" if all k-mers on the unitig are associated with the same set of colors. My question is: how many unitigs in the pangenome graph are color consistent? Is it true that a unitig can only be color inconsistent when it involves an end of a contig? I couldn't think of a counter example. If my statement is true, then all unitigs should be color consistent in a genome graph constructed from circular genomes. Colored unitigs are more useful and handy than colored k-mers.

**Authors Response**

**Point-by-point responses to the reviewers’ comments:**

*The manuscript has been updated to indicate how many unitigs are color consistent in the graph coloring experiment, we found that 98.72% of contigs are color consisten. When the colored and compacted de Bruijn graph is built from assembled genomes, it is true that a unitig can be color inconsistent when it involves the beginning/end of a contig. It can also be color inconsistent if a contig from an input assembled genome is a substring of a unitig from the graph. This could occur, e.g. when a bacterial chromosome is assembled into two disjoint pieces, whereas the break might not be present in another species. When the two assemblies are colored, the missing k-mers in the first would be present in the contig, but the colors would reveal the break. Colored k-mers are more flexible than colored unitigs and might be more useful in the case of colored graphs built from short read data rather than assembled genomes.*
